# A specialist’s audit of aggregated occurrence records: An ‘aggregator’s’ perspective

**DOI:** 10.3897/zookeys.305.5438

**Published:** 2013-05-30

**Authors:** Lee Belbin, Joanne Daly, Tim Hirsch, Donald Hobern, John La Salle

**Affiliations:** 1 Atlas of Living Australia, CSIRO Ecosystem Sciences, GPO Box 1700, Canberra, ACT 2601; 2 Blatant Fabrications, Carlton, Tasmania; 3 CSIRO, Strategic Advisor, Environment Group, GPO Box 1700, Canberra, ACT 2601; 4 Global Biodiversity Information Facility, GBIF Secretariat, Universitetsparken 15, DK-2100 Copenhagen Ø, Denmark

**Keywords:** Australia, occurrence records, data quality, data cleaning, ALA, GBIF, millipede, fitness for use

## Abstract

A recent ZooKeys’ paper (Mesibov, 2013: http://www.pensoft.net/journal_home_page.php?journal_id=1&page=article&SESID=df7bcb35b02603283dcb83ee0e0af0c9&type=show&article_id=5111) has highlighted data quality issues in aggregated data sets, but did not provide a realistic way to address these issues. This paper provides an aggregator’s perspective including ways that the whole community can help to address data quality issues. The establishment of GBIF and national nodes (national aggregators) such as the Atlas of Living Australia (ALA) have integrated and exposed a huge diversity of biological observations along with many associated issues. Much of the admirable work by Mesibov (2013) was enabled by having the data exposed.

Data quality, one of the highest priorities for GBIF, the national nodes and other aggregators, depends on both automatic methods and community experts to detect and correct data issues. Not all issues can however be automatically detected or corrected, so community assistance is needed to help improve the quality of exposed biological data. We do need to improve the infrastructure and associated processes to more easily identify data issues and document all changes to ensure a full record is permanently and publicly available.

## Introduction

Mesibov’s paper (Mesibov 2013) was a welcome audit and critique of millipede records in three sources: Millipedes of Australia (MoA; http://www.polydesmida.info/millipedesofaustralia/); the Global Biodiversity Information Facility (GBIF; http://www.gbif.org/) and the Atlas of Living Australia (ALA; http://www.ala.org.au/), the Australian node for GBIF. This paper identified a range of known issues about data that the authors and broader biodiversity informatics community would like to see addressed as efficiently as possible.

The establishment of GBIF in 2001 seeded a wide range of positive national and international developments. For example, GBIF strongly supported Biodiversity Information Standards (Taxonomic Databases Working Group) in the development of standards such as Darwin Core and ABCD (http://www.tdwg.org) that are required for the efficient communication of biological records among agencies. A wide range of publications has also been commissioned by GBIF, a number of which address data quality issues (e.g., Chapman 2005a and 2005b; Costello et al. 2012). The requirement of GBIF for participating countries to set up national nodes has also focused attention on the status of national biological records. As with any advances however, there is potential for misinterpretation of aims and outcomes coupled with an expectation that agencies such as GBIF will run before they can crawl – data issues are being addressed but not as fast as the community would wish.

The following points need to be considered in addressing data issues:

1. Data quality and the ability to clean and correct data are the responsibility of the community and cannot be assigned to any one agent in the process. There is the need to seamlessly integrate expert knowledge and automated processes.

2. Herbarium or museum records, or even a single collector’s records, are all aggregations of records taken at different times and by different collectors. In the digital world, the flow of biological observations can go from observer to end user through multiple digital aggregators. Mesibov (2013) too is a data aggregator of Australian millipedes. At any node in the flow, errors can be detected, introduced or addressed.

3. Data should be published in secure locations where they can be preserved and improved in perpetuity. This means moving beyond storage of data by individuals, or on stand-alone computers, or even in institutions that do not have a strategy for enduring digital storage and access.

4. We need an effective way to support experts so all amendments form part of a persistent digital knowledge about species. Talented and committed individuals can make enormous progress in error detection and correction (as seen in Mesibov 2013) but how do we ensure that when an individual project like that on millipedes ceases, the data and all associated work are not lost? How do we achieve this in situations when different experts are able to contribute to different aspects of correcting the same data (some working on fine-scale georeferencing, some on taxonomy, etc.)?  All of this implies standards in capturing and linking this information and maintaining the data with all amendments documented. To achieve this the biodiversity research community needs to be motivated and empowered to work in a collaborative fashion.

5. We need to move from a mind-set based on historical approaches that managed paper-based information to one where all relevant information is generated, managed and curated in a fully interlinked form. We need to build a comprehensive digital global knowledgebase for biodiversity to replace our paper-based knowledgebase.

6. Addressing data errors will involve the ‘aggregators’ improving their ability to detect and correct errors. These organisations have a responsibility to deliver automated mechanisms wherever possible and to facilitate new processes and tools that will support the other aspects listed above.

## Discussion

‘Data quality’ is one of the highest priorities for agencies such as GBIF and the ALA, as well as one of the main concerns of users of data (see Otegui et al. 2013). In both agencies, considerable resources have been assigned to help identify and address errors within data records. For example, the ALA appointed a specialist for a year to assist in establishing tools and methods to help identify data issues. This work is ongoing.

While data quality is of the highest concern, published data have many different uses. Data may not need to be 100% accurate for them to have utility. Quality issues affecting some users may be of secondary or no importance to others. For example, a locational inaccuracy of 20km on a record will not invalidate its use with regional or continental scale studies. Access to information on a type specimen is likely to be of value even if georeferences are incomplete or incorrect. The term ‘fitness for use’ may therefore be more appropriate than ‘data quality’ in many circumstances. This is not an excuse to ignore errors, but recognition that effective use depends on knowledge of the data involved.

The goal of the aggregators is to address known problems in data, to understand how much confidence is appropriate in each element of each record and to enable users to filter data based on these confidence measures. The philosophy of most of the aggregators is therefore to flag potential issues, correct what is obviously correctable and expose the flag rather than hide or remove the associated record.

One of the most powerful outcomes of the publishing of digital data is that inherent problems in legacy data are revealed despite the concerted work of dedicated taxonomists over decades or longer. Data are highly variable and not always reliable. Exposing data provides the opportunity for the community to detect and correct errors. Indeed, much of the admirable work achieved by Mesibov (2013) was enabled by having data exposed by the institutions concerned.

As noted by Mesibov (2013), querying and correcting records with a museum often required an email or a phone call. However, museums and herbaria do not always have the infrastructure that agencies like GBIF and the ALA have for interrogation and correction of records. GBIF and the ALA’s expertise is in the area of information technology and biodiversity informatics and better placed to provide online infrastructure support.

GBIF seeks to stimulate best practice in biodiversity data publishing and this includes addressing data quality at the source. There are a range of freely available tools, documents and training programs covering issues such as data cleaning (see http://www.gbif.org/orc and http://www.gbif.org/participation/training/). GBIF also emphasises the value of comprehensive metadata including the option of peer reviewed ‘data papers’ for enhancing the fitness for use of published data (see http://www.gbif.org/communications/news-and-events/showsingle/article/new-incentive-for-biodiversity-data-publishing).

Specialist domain expertise is required to detect and correct a range of error types, as is shown by Mesibov’s (2013) expertise with Australian millipedes. The ALA and GBIF do not generally have this type of expertise. They do however have expertise to build infrastructure that enables integrated data to be openly discovered and where errors are more likely to be exposed. Agencies such as GBIF and the ALA are also in a good position to provide infrastructure and processes that help to address data issues. An example of the quality controls undertaken by the ALA can be seen in Table 1. Other examples from GBIF and national nodes are given below and in the Appendix.

**Table 1.** Example of automated data checks within the Atlas of Living Australia.

**#**	**FLAG**	**% of records**
1	Missing coordinate precision	90.2%
2	Geodetic datum assumed WGS84	44.4%
3	Decimal Latitude Longitude Converted	27.6%
4	Unrecognized geodetic datum	21.1%
5	Coordinate uncertainty not specified	18.6%
6	Possible duplicate record	8.4%
7	Invalid collection date	8.0%
8	No collection date supplied	4.1%
9	Coordinate uncertainty not valid	2.6%
10	Habitat incorrect for species (user flagged issue category)	2.4%
11	Name not in national checklists	2.3%
12	Basis of record not supplied	2.1%
13	Altitude value non-numeric	2.0%
14	Name not in any national or international checklists	1.1%
15	Suspected outlier (user flagged issue category)	1.0%
16	Type status not recognized	<1%
17	Basis of record badly formed	<1%
18	Coordinates don’t match supplied state	<1%
19	Supplied country not recognized	<1%
20	Image URL invalid	<1%
21	Supplied coordinates are zero	<1%
22	Collection code not recognized	<1%
23	Min and max depth reversed	<1%
24	Unparseable verbatim coordinates	<1%
25	Coordinates derived from verbatim coordinates	<1%
26	Latitude is negated	<1%
27	Depth value non-numeric	<1%
28	Outside expert range for species	<1%
29	Longitude is negated	<1%
30	Min and max altitude reversed	<1%
31	Coordinates were transposed	<1%
32	Decimal Lat/Long calculated from easting-northing (grid reference)	<1%
33	Supplied coordinates centre of state	<1%
34	Coordinate precision and uncertainty transposed	<1%
35	Coordinates are out of range for species	<1%
36	Decimal Lat/Long calculated from Easting Northing Failed	<1%
37	Coordinates centre of country	<1%
38	Geospatial issue (user flagged issue category)	<1%
39	Day and month transposed	<1%
40	Depth out of range	<1%
41	Taxon misidentified (user flagged issue category)	<1%
42	Taxonomic issue (user flagged issue category)	<1%
43	Temporal issue (user flagged issue category)	<1%
44	Altitude out of range	<1%

There are two types of data quality issues, those that can be detected without domain specific taxonomic expertise and those that require domain specific taxonomic expertise for detection. Correction of detected issues may or may not require domain specific expertise (see Table 2). Obviously GBIF and the ALA have many tools that can help address Type 3 and 4 cases. For example, an observation of a terrestrial species that occurs in a marine environment would be Type 3 if the true location of the observation was known and Type 4 if not.

**Table 2.** A two-way decision table of issue detection versus correction.

		Domain specific expertise required to address issue?	
		**Yes**	**No**
Domain specific expertise required to detectissue?	**Yes**	Type 1	Type 2
	**No**	Type 3	Type 4

Many of the issues that Mesibov (2013) raised fall into Type 1, for example “Provider G supplied 67 records with the wrong species names, i.e. incorrect specimen identifications. I supplied correct identifications for these records in 2005…”. ‘Aggregators’ such as GBIF and the ALA should strive to address all Type 4 (e.g., a transposition of longitude and latitude) and highlight Type 3 issues (e.g., a marine species on land). We would assume that Type 2 examples would be rare: The domain specific taxonomic expertise required to detect the issue would also be able to make correction possible (Type 1).

A more fundamental issue is that most biodiversity data today are managed and published through a wide range of heterogeneous databases and processes. Consistency is required for guaranteed, stable, persistent access to each data record and in establishing standardised approaches to registering and handling corrections. Any aggregator has a key role in addressing this challenge but ultimately it will depend on widespread changes in the culture of biodiversity data management.

Manual checking as demonstrated by Mesibov (2013) is time consuming yet necessary for a range of issues where automated checking cannot be guaranteed to find and correct issues. GBIF and the ALA do have an extensive suite of automated checks (‘rules set’, see Table 1 and https://docs.google.com/spreadsheet/ccc?key=0AjNtzhUIIHeNdHJOYk1SYWE4dU1BMWZmb2hiTjlYQlE#gid=0). As pointed out by Mesibov (2013) however, they do not always work, but such checks and corrections remain a cost-effective and necessary step. A more robust rule set is in continual development and Mesibov’s paper will help with this. Contributions to these rules by the community would be appreciated.

The ALA has also established a sophisticated annotations service that enables crowd sourcing to detect and correct data errors (see http://www.ala.org.au/blogs-news/annotations-alerts-about-new-annotations-and-annotations-of-interest/). Such detected issues, with potential corrections are returned to the data provider. This is the second flow model of Mesibov (2013), viz.

specialist → GBIF, ALA → data providers → OZCAM → GBIF, ALA

This model as well as Mesibov’s first model, viz.,

specialist → data providers → OZCAM → GBIF, ALA

will, as noted above, depend on the resources of the data provider. Users of the GBIF data portal can also report errors which are relayed by email directly to the original data publishers. Sadly, however, museums and herbaria may not have staff resources to update their databases when issues are reported.

There is no doubt that communication between the relevant taxonomic domain experts to correct problems will be most efficient. Errors will remain if that expertise no longer resides with the data provider, or that provider doesn’t have the resources.

Data providers have diverse expectations. Some data providers encourage the ALA to make corrections to the provider’s records (for provider and ALA). Other data providers would withdraw their support if similar changes were attempted on their data by the ALA. Feedback from the ALA to a data provider may result in immediate corrections (and data propagation) while in other cases, the provider has no resources to resolve an issue. There is no single process here that will work effectively in all circumstances. We do however take Mesibov’s paper as a prod to seek best current practice among providers and aggregators to improve data quality.

At the global level, GBIF’s current Work Programme includes provision for an upgraded data portal, being rolled out progressively from 2013, to also support an annotations service. The Botanical Garden and Botanical Museum Berlin-Dahlem (BHBM), which hosts the GBIF node for Germany, has prototyped a generic annotations system for biodiversity data known as Annosys (see http://wiki.bgbm.org/annosys/index.php/Main_Page).

## Conclusions

Agencies such as the ALA and GBIF enable observations to be recorded directly to their systems. These records are reviewed before being ‘published’, but the ALA and GBIF are not the data provider and therefore cannot assume responsibility for these records; disclaimers are therefore necessary.

There is however full agreement that aggregators such as GBIF and the ALA have a responsibility to detect and where possible address data issues with the data provider’s permission and support. There is no doubt that data errors are best addressed through collaboration between all relevant agencies. GBIF itself and projects such as CReATIVE-B (http://creative-b.eu) are in a good position to facilitate such collaboration on the development of broadly agreed tools and processes.

Agencies such as GBIF and the ALA have the mandate to expose a large volume of data records in a systematic format. The aggregation process can itself result in an enhanced ability to identify errors. For example the ALA uses a tool that examines the environmental envelope associated with a species to help identifier environmental outliers. This process would not be possible without a critical mass of observations.

What ideally is needed is an environment created by agencies such as GBIF and the ALA that *efficiently* enables

1. Exposure of errors

2. Discussion of the errors

3. Addressing errors directly in *all relevant locations*

No such environment currently exists. Progress will be limited while the underlying culture of data publishing and data management does not support stable, long-term reference to each data record and community-based curation of those data in a way that ensures that each act of correcting any aspect of any data element is not lost but contributes to the development of a global digital biodiversity knowledgebase. However, it will also require a fundamental change in data management at the institutional and personal level. All data needs to be published in stable locations where it can be preserved and improved in perpetuity and the biodiversity research community needs to be motivated and empowered to do its work in an online collaborative way. A recent paper sponsored by GBIF (Costello et al. 2012), among other things, applauds data publishing but suggests that data quality could be improved by peer review.

## Appendix

Examples of data quality routines that are in place or under development by GBIF national nodes and other data aggregators are included here to indicate that data quality is both a high-priority and a rapidly developing area in our community.

**The Atlas of Living Australia (ALA)** is covered above.

**GBIF Norway** checks all scientific names against the Norwegian Species Name Database (Artsnavnebasen). Names that do not match are collected into a list and returned to the data owner, but unless anything is obviously wrong, the record is still served to the GBIF network. The coordinates of all georeferenced records are converted to WGS84 and matched against a country bounding box and a municipality bounding box. Coordinates that do not match the country and municipality bounding boxes are collected and returned to the data owner. Country names, county names and municipality names are matched against lists of approved names. Dates are validated, and for certain datasets they are matched against a known start date and end date.

8.6 million citizen science records served from the Norwegian Biodiversity Information Centre’s species observation portal (http://www.artsobservasjoner.no/) are validated by other citizens and by a scientific expert panel. Only valid names from the official Norwegian list of taxa present in Norway are shared with GBIF, pending validation by a scientific name committee.

**The United Kingdom’s National Biodiversity Network (NBN)** is engaging with experts to develop rules to help screen data – for example relating to known distributions or migration periods. If a record falls outside these rules it is flagged for further scrutiny – not implying that it is wrong but ‘interesting’ in that it changes our understanding of the species if proven correct. The NBN has developed a desktop tool called Record Cleaner (http://www.nbn.org.uk/record-cleaner.aspx). This tool enables a data publisher to access the source (for example a database or spreadsheet) for screening, thus identifying potential issues before publication. Record Cleaner both validates (checking syntax) and verifies (checking against expert rules). The rules are being integrated into NBN’s online recording service, alerting wildlife recorders to ‘interesting’ records at the point of entry, helping to detect problems early and indicating that additional information or evidence may be required.

**GBIF Spain** places particular emphasis on data quality, for example operating a ‘Biodiversity Data Quality Hub’ (http://www.gbif.es/BDQ.php) bringing together tools for error detection and validation of biodiversity data (Otegui et al. 2013). These tools include the Darwin Test tool (http://www.gbif.es/darwin_test/Darwin_Test_in.php) developed by GBIF Spain to validate data entered in Darwin Core format. As a complement to this tool, the node provides an ‘apparent quality index’ (http://www.gbif.es/ICA.php) applied to all datasets published by Spanish participants, which enables a record to be kept of increases in quality as well as quantity of biodiversity data published from institutions in Spain. GBIF Spain’s work on data quality has formed the basis of a number of training workshops and online learning modules involving several countries in Latin America, Africa and Europe (see for example http://www.gbif.org/orc/?doc_id=2914&l=en, http://www.gbif.org/participation/training/events/training-event-details/?eventid=203, http://www.gbif.es/formaciondetalles.php?IDForm=109, http://elearning.gbif.es/AContent/home/index.php).

**GBIF Colombia (Sistema de información sobre biodiversidad de Colombia)** has focused on data quality within the country’s biological institutions as part of a recent overhaul of its data publishing and access service (see www.sibcolombia.net). The node is in the process of implementing several tools and protocols on data cleaning, including adaptation of some of the tools developed by GBIF Spain and a customized taxonomic and geographic validation tool for biodiversity records in Colombia and neighbouring countries (see http://code.google.com/p/verificacion-registros-biologicos/). The node is also working on an online data validator using Google Docs.

**CRIA Brazil, (the Reference Center on Environmental Information**) is a not-for-profit, non-government organization whose aim is to contribute towards a more sustainable use of Brazil’s biodiversity through the dissemination of high quality information and education. CRIA has had an extensive and illustrious history in the development of tools for analysing and cleaning biological records, see http://splink.cria.org.br/tools?criaLANG=en.
